# Improving the quality of childbirth services in Zambia through introduction of the Safe Childbirth Checklist and systems-focused mentorship

**DOI:** 10.1371/journal.pone.0244310

**Published:** 2020-12-30

**Authors:** Sandra Mudhune, Sydney Chauwa Phiri, Marta R. Prescott, Elizabeth A. McCarthy, Aaron Banda, Prudence Haimbe, Francis Dien Mwansa, Angel Mwiche, Andrew Silumesii, Kabamba Micheck, Hilda Shakwelele, Margaret L. Prust

**Affiliations:** 1 Clinton Health Access Initiative, Lusaka, Zambia; 2 Clinton Health Access Initiative, Boston, MA, United States of America; 3 Ministry of Health, Lusaka, Zambia; University of Michigan, UNITED STATES

## Abstract

Although strong evidence exists about the effectiveness of basic childbirth services in reducing maternal and newborn mortality, these services are not provided in every childbirth, even those at health facilities. The WHO Safe Childbirth Checklist (SCC) was developed as a job aide to remind health workers of evidenced-based practices to be provided at specific points in the childbirth process. The Zambian government requested context-specific evidence on the feasibility and outcomes associated with introducing the checklist and related mentorship. A study was conducted on use of the SCC in four facilities in Nchelenge District of Zambia. Observations of childbirth services were conducted just before and six months after the introduction of the intervention. Observers used a structured tool to record adherence to essential services indicated on the checklist. The primary outcome of interest was the change in the average proportion of essential childbirth practices completed. Feedback questionnaires were administered to health workers before and six months after the intervention. At baseline and endline, 108 and 148 pause points were observed, respectively. There was an increase from 57% to 76% of tasks performed (p = 0.04). Considering only these cases where necessary supplies were available, health workers completed 60% of associated tasks at baseline compared to 84% at endline (p<0.01). Some tasks, such as taking an infant’s temperature and hand washing, were never or rarely performed at baseline. Feedback from the health workers indicated that nearly all health workers agreed or strongly agreed with positive statements about the intervention. The performance of health workers in Zambia in completing essential practices in childbirth was low at baseline but improvements were observed with the introduction of the SCC and mentorship. Our results suggest that such interventions could improve quality of care for facility-based childbirth. However, national-level commitment to ensuring availability of trained staff and supplies is essential for success.

**Trial registration** Clinical Trials.gov (NCT03263182) Registered August 28, 2017

This study adheres to CONSORT guidelines.

## Background

High-quality health services and the provision of basic, evidence-based services in the childbirth and immediate post-partum period can reduce maternal and newborn mortality. Even though 67% of women in Zambia deliver in a health facility [1], Zambia has seen limited progress in improving maternal and neonatal outcomes. Recent analyses have shown that worldwide, a higher number of excess deaths occur each year from all causes due to poor-quality care versus non-utilization of or lack of access to services [2]. In Zambia and many similar settings, barriers such as insufficient staffing and skills, limited availability of equipment, drugs, and supplies, and an ineffective referral system for women and infants needing high-level care can inhibit the provision of quality health services [3]. In this context, comprehensive interventions are needed to address these gaps and ensure that resources are effectively used to enhance patient outcomes and experiences [4].

The Safe Childbirth Checklist (SCC) was developed by the World Health Organization in 2010 [5] to support health workers to perform essential, evidence-based practices as a part of childbirth services. The SCC is designed to help birth attendants remember to conduct 29 essential birth practices at four critical pause points (PPs) in the delivery process, including: on arrival (pause point 1), just before pushing (pause point 2), within one hour of delivery (pause point 3), and before discharge (pause point 4). Numerous published studies concerning the introduction of the SCC in specific country contexts have demonstrated that it can be effective in influencing health worker behavior on specific tasks [6–15].

At the same time, evidence shows that the SCC alone may not produce reductions in mortality, even when introduced with basic, skills-based mentorship. In particular, a recent large-scale cluster-randomized trial in India called the BetterBirth trial found that performance of the practices recommended by the SCC increased significantly in the intervention group, however there was no significant difference in the intervention and control groups on a composite outcome of perinatal death, maternal death, or maternal severe complications within seven days after delivery [16]. The authors of this study hypothesized that although performance of essential birth practices improved, the levels of adherence to the full range of essential practices may not have been sufficient to affect patient outcomes. The program did not explicitly address systems issues related to poor labor ward organization and management, weak supply chains, and inadequate documentation and data use; and these gaps may have influenced the ability of health workers to carry out the recommended practices. The BetterBirth program in India has been the largest effort to date to roll out and evaluate the impact of the SCC.

Our study aimed to build on previous evidence suggesting that introduction of the SCC alone is not enough to change how childbirth services are delivered. We aimed to measure the change in health worker practices following the introduction of the SCC along with a scalable approach to mentorship that focused on addressing health systems gaps to achieve consistently high adherence to essential birth practices. This evidence was meant to support policy decisions in Zambia about whether and how to introduce the SCC.

## Methods

This study was implemented from July 2017 and April 2018, using a pre-post design. The primary outcome was the change in the average proportion of essential childbirth practices completed. The evaluation focused on 21 observable tasks that should be completed on all mother-infant pairs, regardless of health status. This was measured through observations of childbirth practices in four purposefully selected facilities before and at six months after the introduction of the intervention. Nchelenge District was chosen by the government to be the location for this pilot based on need for support to improve maternal and newborn outcomes. Within Nchelenge District, four facilities were selected based on high demand for services, perceived need for support for quality improvement, and presence of a trained skilled birth attendant to mentor. During the observation periods in these four health facilities, all consecutive deliveries were observed if consent was given.

The SCC was adapted to the Zambian context and introduced along with systems-focused mentorship that aimed to improve not only health worker knowledge and skills but also the availability of supplies and equipment, documentation and data use, and the enabling environment at health facilities in Zambia. The mentorship began with a six-week intensive coaching period at the time of the SCC launch, during which time weekly mentorship was provided. After this period and for the next six months, mentorship visits of two to three days were made monthly by district level teams. Each mentorship visit began with an assessment of clinical competencies of the health workers present as well as systems level gaps related to supplies, documentation, and enabling environment. The assessment was performed using a standardized checklist. After the assessment the mentor and facility staff developed an action plan and worked together to address issues through clinical coaching, training on documentation or environmental issues, and escalation of issues.

For the secondary outcomes related to health workers characteristics and feedback, all health workers in the four study sites were invited to participate. Health worker questionnaires were also administered to understand their feedback on the success and acceptability of the checklist and mentorship. This study adheres to CONSORT guidelines.

A full description of the methods has been published elsewhere [17].

### Statistical analysis

The percentage of tasks completed during each observed pause point was calculated and an average was taken across all observations for each time point. The differences from baseline to endline were examined first using paired, weighted t-tests to account for differences in the standard errors and then using linear regression accounting for clustering at the facility level. As a sensitivity analysis, since not all potential activities within the SCC could be completed due to lack of supplies, the percentage of SCC items completed in each observed pause point was adjusted by removing such activities from the denominators of the calculations. Some tasks, such as early initiation of breastfeeding do not require any outside supplies so these tasks were always included in the denominator. Finally, we examined the proportion of births increased where the 75% of tasks were completed; specifically, we examined if the difference overtime in the average proportion of births where 75% of tasks were completed increased due to the intervention. To this end, we ran individual-level logistic regression, accounting for clustering at the facility level. In relation to health worker surveys, descriptive statistics are provided.

### Ethics approval and consent to participate

Ethical approval was granted by two research ethics committees: Advarra IRB (formerly Chesapeake) where approval was granted on 15 June 2017 (Pro00022097) and ERES Converge IRB of Zambia where approval was granted on 8 July 2017 (2017-May-066). The study also received approval from the National Health Research Authority in Zambia.

All skilled birth personnel were asked to provide written informed consent to be observed at the launch of data collection. For ethical reasons, data collectors were instructed not to interfere with clinical care during their observations unless a patient’s life was in danger, and in such cases to inform the health worker directly.

When a pregnant woman presented at the health facility in labor, the health worker managing the delivery process informed the potential participant that childbirth services were being reviewed in that facility and that a data collector would approach her to ask about observing the services provided during her delivery. Written consent was obtained from all women for their delivery to be observed. The health worker performing the delivery services was asked to act as the witness on the consent form if the woman agreed to participate.

Before participating in the health worker questionnaire, study staff informed potential participants about the purpose of the study, and that their participation would be anonymous and voluntary. Health workers were asked to check a consent box at the top of the questionnaire if they wished to participate.

## Results

For the intervention, a total of 204 individual mentorship sessions were provided for 36 staff members from September 2017 to April 2018 in the four facilities where this evaluation took place. During the evaluation, a total of 108 and 148 pause points were observed across 44 and 66 unique patients at baseline and endline, respectively (Table 1). This resulted in 570 task observations at baseline and 777 task observations at endline. Observers were not present at the facility 24 hours a day, which meant that they missed some pause points for some deliveries, though study protocols dictated that they aimed to capture as many pause points for each delivering mother as possible.

**Table 1:** Observations and patients observed at baseline and endline.

Sample size dimension	Baseline	Endline
Total pause point observations	108	148
Total task observations (of 21 key tasks)	570	777
Unique patients were observed at any pause point	44	66
Patients were observed from Pause point (PP1) through Pause point (PP3)	19	28
Patients were observed from Pause point (PP1) through Pause point (PP4)	12	14

### Performance of essential childbirth practices

**Fig 1:**
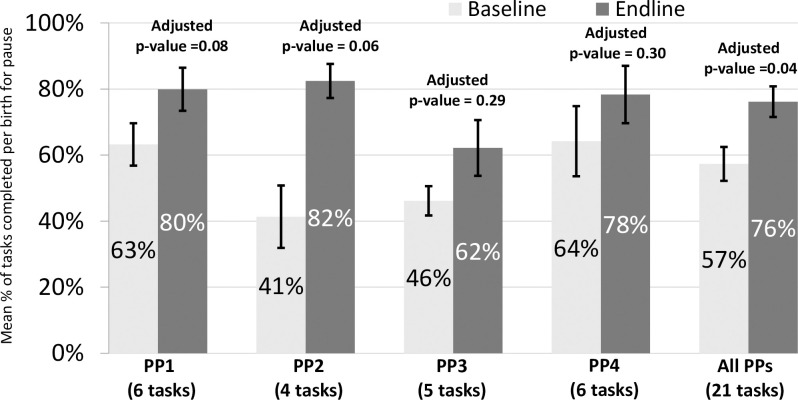
Percentage of tasks performed, by pause point (not controlling for supplies). All adjusted p values in this figure represent regression analyses that control for clustering by facility while the percentages and confidence intervals shown are from adjusted t-tests.

Across all pause points and all facilities, 57% (of 570 task observations) and 76% (of 777 task observations) of the tasks were performed at baseline and endline, respectively (Fig 1). This change in performance of tasks was significant across all observations (p = 0.04) but non-significant for individual pause points due to smaller sample size of task observations at this level.

**Fig 2:**
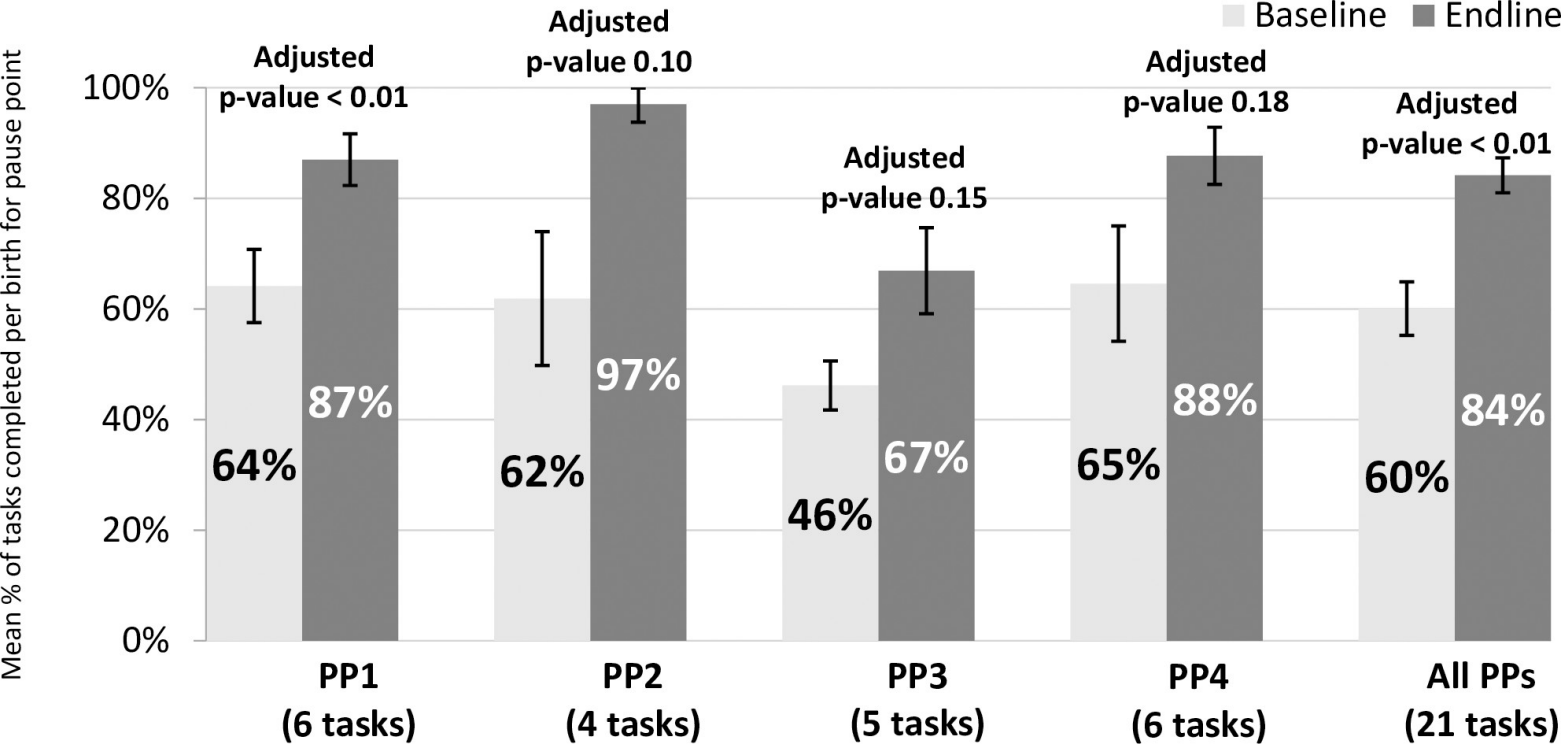
Percentage of tasks performed, by pause point (controlling for supplies). All adjusted p values in this figure represent regression analyses that control for clustering by facility while the percentages and confident intervals shown are from adjusted t-tests.

We also assessed whether tasks were completed successfully only in the cases where the supplies necessary to complete the task were available. Across all pause points, 60% of tasks were completed at baseline compared to 84% at endline (p<0.01). In pause point 1, the change from 64% of tasks completed at baseline to 87% at endline was also significant (p<0.01) (Fig 2).

**Fig 3:**
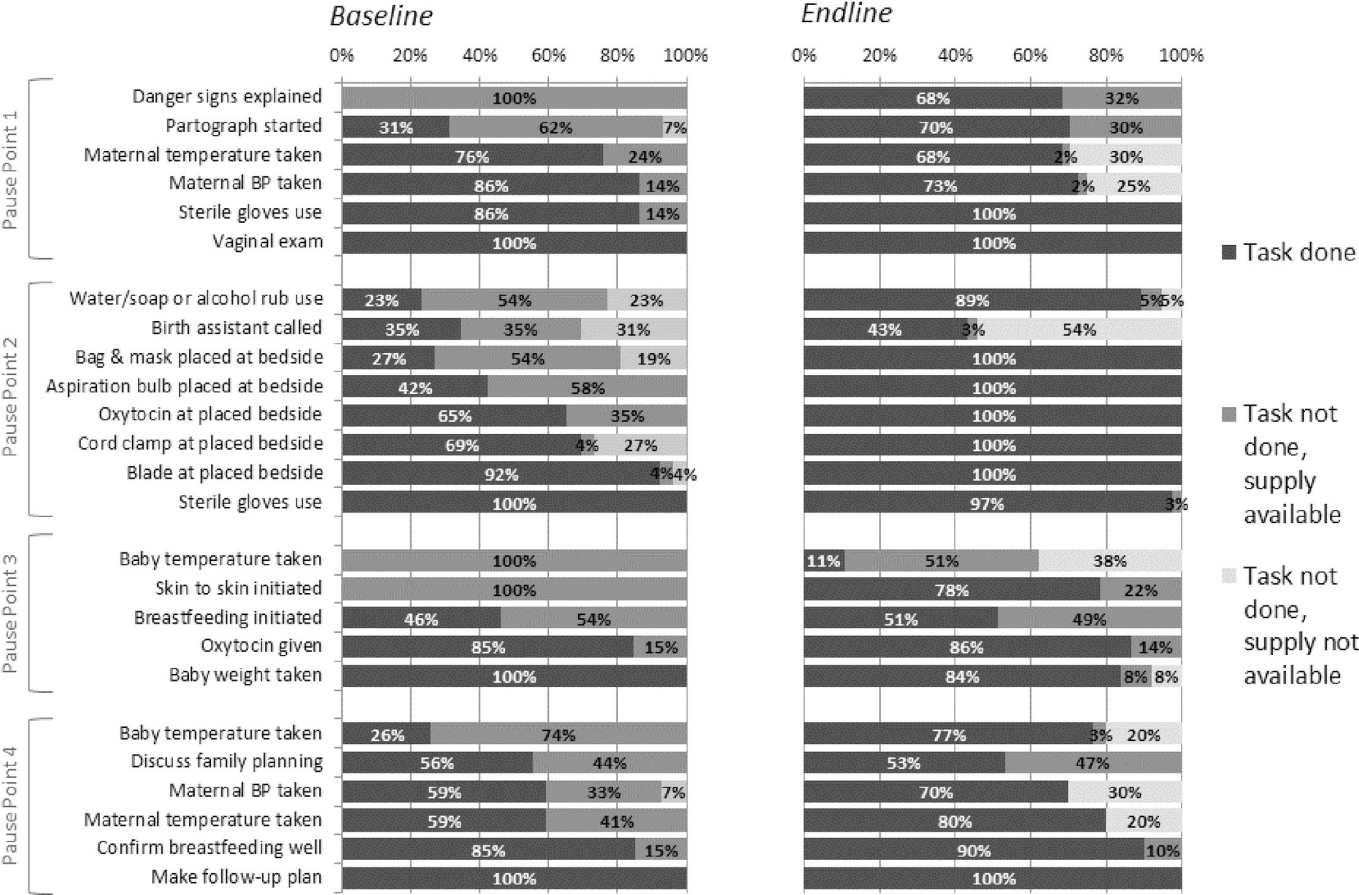
Percentage of deliveries where each task was completed.

There was some variation in the percentage of observed cases in which each task was performed (Fig 3). While some tasks were regularly completed at baseline, other tasks were never or rarely performed, such as taking an infant’s temperature and hand washing. Danger signs were not explained to the mother or care giver and skin-to-skin care was not initiated in any observations even though these tasks do not require any supplies. At endline there was an improvement in completion of individual tasks: 14 of 21 essential childbirth practices were completed in at least 80% of observations or greater, compared with only 9 of 21 practices at baseline. There were no tasks that were never done across all observations at endline (Fig 3). However, there are some tasks that show a decline from baseline to endline, most notably baby weight taken after birth and maternal blood pressure taken at admission. At endline, there were several cases of lack of supply availability (shown in the lightest grey in Fig 3), but in cases where supplies were available, health workers more frequently used those supplies at endline than at baseline. Across all pause point observations, there was a decrease from 39% to 13% in the percentage of observations where the task was not done yet the supply was available; however, the percentage of cases in which the necessary supplies were not available increased from 5% to 11% from baseline to endline (data not shown).

### Performance of essential childbirth practices across full births

In the deliveries that were observed completely from pause point one to four, the minimum percentage of tasks performed in any one delivery was 48% and the maximum was 76% at baseline, while at endline the minimum was 43% and the maximum was 95%. At baseline, 75% or more of the 21 total tasks were completed in only one of twelve fully observed deliveries (8.3%) at baseline compared to 11 of 14 fully observed deliveries (78.6%) at endline.

### Feedback from health workers on the SCC

**Fig 4:**
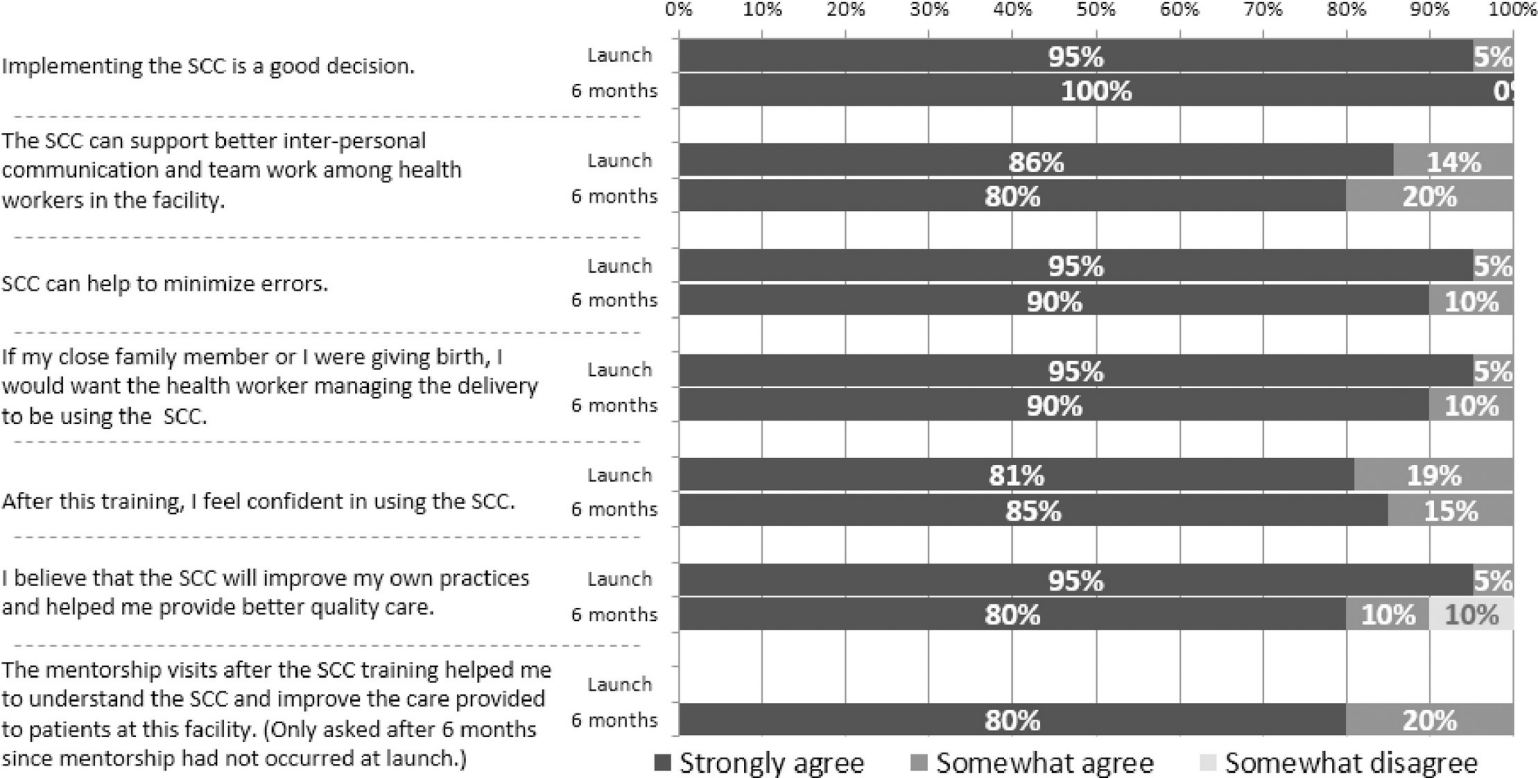
Feedback from health workers on the SCC.

Feedback on the checklist was solicited from the health workers at the four facilities before and after the introduction of the checklist to determine success and acceptability of the checklist and mentorship. There were 21 respondents at baseline and only 20 respondents at endline with the difference attributed to staff changes and staff availability. Participants were asked how much they agreed with a list of statements about the SCC, and 80% of participants strongly agreed with the statements in Fig 4 about the SCC and there were no clear response patterns by facility.

## Discussion

In this study, the SCC and associated mentorship program was successfully implemented in four facilities in Nchelenge District with positive feedback from health workers. From baseline to endline, we observed a significant improvement in adherence to essential birth practices by the health workers considering all tasks across all observed pause points. The percentage of tasks completed increased in each individual pause points, though the changes were non-significant. The change in percentage of tasks completed at baseline and endline was even larger when only considering the tasks for which supplies were available to conduct the tasks. In other words, the percentage of tasks that were not completed increased yet the supply was available decreased, suggesting that use of available supplies improved.

Differences existed across specific tasks in the completion of tasks. For example, at baseline the tasks least likely to be completed were related to checking vital signs (infant temperature and mother temperature and blood pressure), infection prevention and control (hand washing), communication with patients (explaining danger signs, discussing family planning), and skin-to-skin contact. The biggest improvements at endline were seen in relation to explaining danger signs, hand washing, placing supplies at the bedside before birth, and skin-to-skin contact. These results are supported by evidence observed in other contexts [7, 16]. It was noted that health workers almost always referred to the SCC at every pause point, but still did not always complete the tasks being tracked by the observers. This discrepancy may speak to the fact that, despite mentorship, the health workers interpreted the items listed in the SCC differently than intended. Future studies should consider options for ensuring closer alignment between the SCC and observation tools, either by adjusting the observation tool and process or by revising the SCC to make it more task-oriented.

The completion of tasks at baseline in this study in Zambia was 57%, which is somewhat higher than in many studies [13, 14, 16], however, one of the continued challenges at baseline and throughout the study period was limited availability of supplies. Although the mentorship program implemented in this study was designed to address systems gaps, such as lack of supplies, in addition to clinical competency gaps, the percentage of cases in which the necessary supplies were not available increased slightly from 5% to 11% from baseline to endline, suggesting that even in the context of this systems-oriented intervention, supply availability continues to be a challenge in health facilities. In the Zambian context, mentors traditionally focus on clinical skills and knowledge rather than systems improvements. Although the implemented approach attempted to broaden the scope of mentorship beyond the focus on individual skills and knowledge, long-standing practices may be difficult to change.

The feedback from the health workers on the acceptability of the checklist shows that they were agreeable with the introduction of the SCC and saw it as a tool that can support communication, minimize error and improve practice. The Sri Lankan study, from which we adopted our feedback tool, also reported high attitudes of using the checklist and our endline results were similar to their findings (109). A pilot study in nine health facilities in Italy also received positive feedback from health workers with most of them agreeing that the checklist had improved patient safety as well as improving teamwork [11].

Measurement of SCC adherence will need to be part of a successful implementation. Future work, however, should continue to evaluate scale up and true impact of SCC in Zambia and elsewhere on maternal mortality rate (MMR) and neonatal mortality rate (NMR). A randomized trial currently being conducted in Kenya and Uganda on the impact of an SCC and quality improvement intervention on NMR should provide valuable results to this end [15].

### Limitations

Results should be interpreted in light of several considerations should be taken into account when interpreting these results. First, one of the assumptions we made was that adherence to essential practices recorded by an observer reflects normal/routine care at the facility. However, it is possible that the Hawthorne effect influenced our measurements. Since mentors also spent a substantial amount of time at each facility during the intervention period, we assume this would reduce the Hawthorne effect. Additionally, any bias introduced by observation would have been consistent at all time points since the data was collected the same way. The BetterBirth [16] study and a number of previous studies [7, 8, 10, 12–14] have measured SCC adherence through direct observations as well. At the same time, other studies have recorded slightly higher adherence to the SCC based on a design that asks health workers to check off items for each patient [10]. It is unclear whether this higher adherence is due to differing contexts, the influence of the requirement to psychically check items off, or health workers checking off tasks that were not actually completed. However, we felt that direct observations were the best way to obtain accurate information on health worker practices.

During data collection no identifiable information was collected about health workers data to protect their privacy, but this also prevented us from linking observations with mentorship data and conducting any analysis that assesses the relationship between performance and the dose of mentorship received. Future studies may consider collecting information about mentorship sessions received and years of practice of the health workers observed at all time points to contribute to sensitivity analyses. Additionally, it may be useful to consider including measures of facility leadership.

Finally, we did not account for other external factors which may have contributed to the changes seen at the health facilities, and we were not able to include a group of control sites to assess whether any changes occurred in the absence of the intervention due to resource constraints and the lack of available sites that could be matched to those selected. However, the implementers of the intervention did not identify other interventions that may have resulted in the changes in adherence to practices.

## Conclusions

Childbirth is a complex health service to deliver and it can be difficult for the health workers to provide all recommended interventions, particularly in low- and middle-income settings. The performance of health workers, at baseline, on routine, evidence-based interventions for safe childbirth was low–but higher than in many other countries. However, significant improvements were seen after the introduction of the SCC and mentorship. Our results suggest that implementation of the checklist with mentorship could produce broad-based improvement in the quality of care of facility-based childbirth, with quality of care measured as health workers’ consistent adherence to practices, which is a necessary step in effecting improvement in health outcomes. However, for the SCC and associated mentorship to truly be successful, there must be a national-level commitment to ensuring the availability of trained staff and supplies. Although this intervention addressed supplies and equipment at the facility and district level, supply chain issues at higher level continued to make it difficult for health workers to carry out their roles.

## Supporting information

S1 Checklist (DOCX)

S1 Data (DTA)

S2 Data (DTA)

## List of abbreviations

CHAI: Clinton Health Access Initiative, Inc.
CI: Confidence interval
EmONC: Emergency Obstetric and Neonatal Care
MMR: Maternal mortality ratio
MOH: Ministry of Health
NMR: Neonatal mortality ratio
SBA: Skilled birth attendant
SCC: Safe Childbirth Checklist
WHO: World Health Organization
